# Altered Cortical-Striatal Network in Patients With Hemifacial Spasm

**DOI:** 10.3389/fnhum.2021.770107

**Published:** 2021-10-22

**Authors:** Wenwen Gao, Dong Yang, Zhe Zhang, Lei Du, Bing Liu, Jian Liu, Yue Chen, Yige Wang, Xiuxiu Liu, Aocai Yang, Kuan Lv, Jiajia Xue, Guolin Ma

**Affiliations:** ^1^Department of Radiology, China-Japan Friendship Hospital, Beijing, China; ^2^Peking University China-Japan Friendship School of Clinical Medicine, Beijing, China; ^3^Department of Neurosurgery, China-Japan Friendship Hospital, Beijing, China; ^4^Department of Ultrasound Diagnosis, China-Japan Friendship Hospital, Beijing, China; ^5^Beijing Laboratory of Biomedical Materials, Beijing University of Chemical Technology, Beijing, China

**Keywords:** hemifacial spasm (HFS), striatum, functional connectivity, motor disorder, resting-state fMRI

## Abstract

**Objective:** Hemifacial spasm (HFS) is a kind of motor disorder, and the striatum plays a significant role in motor function. The purpose of this study was to explore the alterations of the cortical-striatal network in HFS using resting-state functional magnetic resonance imaging (fMRI).

**Methods:** The fMRI data of 30 adult patients with primary unilateral HFS (15 left-side and 15 right-side) and 30 healthy controls were collected. Six subregions of the striatum in each hemisphere were selected for functional connectivity (FC) analysis. One-sample *t-*test was used to analyze the intragroup FC of the HFS group and the control group. Two-sample *t*-test was used to compare the difference of FC between the two groups. The correlation between the abnormal FC and severity of HFS was evaluated by using the Spearman correlation analysis.

**Results:** Compared with the controls, the striatal subregions had altered FC with motor and orbitofrontal cortex in patients with HFS. The altered FC between striatal subregions and motor cortex was correlated with the spasm severity in patients with HFS.

**Conclusion:** The FC of the cortical-striatal network was altered in primary HFS, and these alterations were correlated with the severity of HFS. This study indicated that the cortical-striatal network may play different roles in the underlying pathological mechanism of HFS.

## Introduction

Hemifacial spasm (HFS) is a syndrome of involuntary contraction of facial muscles innervated by ipsilateral facial nerves (Palacios et al., 2008), which can gradually affect facial expressive muscles and platysma muscles (Lu et al., 2014). Primary HFS is believed to be caused by vascular compression of the facial nerve at its root exit zone (Hermier, 2018), but the central mechanism is still not clear. Studies have found that depression and anxiety are more common in patients with HFS (Huang et al., 2009; Rudzińska et al., 2012). Striatum plays a prominent role in modulating motor activity and higher cognitive function (Rosen and Williams, 2001). However, the exact neural mechanism of the striatum in the regulation of motor in patients with HFS still remains unexplored. Early identification of functional changes in the cortical-striatal loop of patients with HFS can help to understand disease pathogenesis and achieve early diagnosis as well as effective treatments. This study was aimed to investigate the altered cortical-striatal network in patients with primary HFS, using resting-state functional magnetic resonance imaging (fMRI).

As part of the extrapyramidal system, the striatum is integral to the motor, cognitive, and emotion regulation functions (Di Martino et al., 2008). The subregions of the striatum, such as putamen, caudate, and ventral striatum, also are associated with different brain functions. Previous anatomical and neuroimaging studies of the striatum have shown that the putamen mainly receives projections from the sensorimotor cortex, and the caudate receives projections from the associated cortex, while the ventral striatum receives projections from the medial prefrontal cortex, orbitofrontal cortex (OFC), and limbic system (Lehericy et al., 2004; Draganski et al., 2008; Choi et al., 2012).

Based on these facts, striatum would be able to exhibit profound influences in motor disorders. For example, as one part of the striatum, putamen can regulate the amplitude and velocity of muscle contraction *via* the cortical-striatal loop and the dopamine system, and therefore plays a significant role in some motor disorders, including Parkinson’s disease and Huntington’s disease (Grillner et al., 2005; Loonen and Ivanova, 2013). The abnormal functional connectivity (FC) between the striatum and the motor cortex was also found in patients with motor disorders (Hacker et al., 2012; Unschuld et al., 2012; Luo et al., 2014). In Parkinson’s disease, after the substantia nigra degeneration, the content of dopamine in the striatum is also decreased (Schroeder et al., 2020), and in addition to the abnormal striatum-substantia nigra loop, the cortex-striatum loop may also be abnormal (Helmich et al., 2010). These results further highlight the significance of the striatum in the development of motor disorders. Besides, the researchers have used FC analysis to study the function of striatal subregions in healthy people (Di Martino et al., 2008), patients with Parkinson’s disease (Helmich et al., 2010; Hacker et al., 2012), depression (Gabbay et al., 2013; Felger et al., 2016), autism (Padmanabhan et al., 2013), and obsessive-compulsive disorder (Harrison et al., 2009), and the mechanisms of central alterations in different subregions of the striatum have been revealed.

Current resting-state fMRI studies of HFS are limited, and the results are diverse. The regional homogeneity (ReHo) index has been used in most previous studies to indicate time-domain coherence between neighboring voxels in the brain. Researchers have found ReHo abnormalities in the motor cortex, frontal lobes, and cerebellum in patients with HFS (Tu et al., 2015; Wei et al., 2015; Lu et al., 2018), and no ReHo alterations in the striatum, probably due to the small sample size and the different meanings between the analysis indexes, i.e., ReHo and FC. However, one study reported that patients after infarction of the caudate, one of the subregions of the striatum, presented with HFS, suggesting that the striatum may play an important role in the development of HFS (Arunabh, Jain and Maheshwari, 1992). In addition, another study on facial nerve palsy found altered FC between the striatum and motor cortex after facial muscle paralysis, further affirming the relationship between the striatum and facial muscle movement (Song et al., 2017). To the best of our knowledge, there are few earlier studies on HFS using the FC analysis method. One study showed abnormalities in FC between the thalamus and parietal cortex in patients with HFS (Niu et al., 2020), but it did not explore the central changes of the striatum in this dyskinesia. HFS is a kind of facial movement disorder, it is not clear that how the striatum regulates the motor function of patients with HFS before and after the onset of its symptoms, and in this study, we focused on striatal function in patients with HFS.

In this study, we performed FC analysis of 12 striatal subregions in patients with HFS. We hypothesized that in patients with chronic primary HFS, the FC between the striatum and motor cortex and FC between striatal subregions will be changed. Since long-term HFS may result in psychological problems, such as depression and anxiety (Bao et al., 2015), we also hypothesized that the FC between the striatum subregions and the emotion-related cortex will be altered in patients with HFS. Furthermore, we explored the relationship between altered FC and clinical characteristics in patients with HFS.

## Materials and Methods

### Subjects

A total of 60 subjects were selected from a total of 64 participants, and 4 subjects (3 patients with HFS and 1 healthy control) were excluded from the analysis due to excessive head motion (translational movement > 2 mm or rotation > 2°). Then, 30 patients with HFS (15 left-side HFS, 15 right-side HFS, 12 men, 18 women, age 48.87 ± 10.61 years) were enrolled from 2017 to 2019 in the Department of Neurosurgery, China-Japan Friendship Hospital. HFS was diagnosed by two experienced neurologists based on clinical symptoms and history. The severity of HFS was assessed using the Cohen spasm scale (0–4 scores, with higher scores indicating more severe spasm) (Cohen et al., 1986). The inclusion criteria for patients were as follows: (1) adult patients with primary unilateral HFS, (2) without craniocerebral lesions and mental disorders, no use of psychotropic drugs, and (3) being right-handed. The exclusion criteria for patients were as follows: (1) with bilateral HFS, (2) having contraindications to MRI examination, and (3) with excessive head motion. Notably, 30 age-, sex-, education-matched healthy controls (12 men, 18 women, age 47.63 ± 13.29 years) were recruited from the society. The inclusion criteria for healthy controls are as follows: (1) aged 18 years old or above, (2) absence of neurological and mental disorders, and (3) being right-handed. The demographic and clinical characteristics of participants are shown in Table 1. This study was approved by the Ethics Committee of our hospital, and all subjects have given informed consent before the experiment.

**TABLE 1:** Demographic and clinical characteristics of participants.

Variable	HFS (*n* = 30) (mean ± SD)	HC (*n* = 30) (mean ± SD)	Two-sample *t* test
			***P* value**
Sex (male/female)	12/18	12/18	−
Age (years)	48.87 ± 10.61	47.63 ± 13.29	0.693
Education (years)	11.57 ± 4.24	13.50 ± 4.35	0.087
Duration (years)	5.70 ± 5.24	N/A	−
Cohen spasm severity (scores)	2.57 ± 0.73	N/A	−

*HFS, hemifacial spasm; HC, healthy control; SD, standard deviation.*

### Magnetic Resonance Imaging Data Acquisition

The experiment was carried out on the 3.0 T MRI scanner (GE, Discovery MR750, Milwaukee, United States) with an 8-channel phased-array head coil. The resting-state fMRI with a single-shot gradient recalled echo-planar imaging sequence was performed with the following recipe. The repetition time (TR) was 2,000 ms, while the echo time (TE) was set to 30 ms. The slice thickness was chosen to be 3.5 mm with a spacing of 0.7 mm. The matrix of the image was 64 × 64, while the field of view (FOV) was 224 mm × 224 mm. The flip angle was 90°, and the number of excitations (NEX) was set to 1. A total of 8 min were consumed for each data with 34 slices and 240 time points. T2WI scan was used to exclude the cerebral organic lesions. 3D T1WI anatomic images were reconstructed using three-dimensional fast spoiled gradient-echo sequences (3D FSPGR), and the TR of which was 6.7 ms, while the TE was set to minimum full. The matrix was changed to 256 × 256 with a FOV of 256 mm × 256 mm. Furthermore, the slice thickness was chosen as 1.0 mm, while the NEX remained to be 1.

### Data Preprocessing

To unify the affected side of the patients, the T1WI and fMRI data with left HFS (15 cases) and matched controls (15 cases) were flipped from left to right before preprocessing (Song et al., 2017). In this study, “right” was defined as the ipsilateral side, and “left” was defined as the contralateral side for the flipped data. The preprocessing was conducted using the software of Data Processing Assistant for Resting-State fMRI (DPARSF) (Yan et al., 2016) in the following steps. First, the DICOM data were converted to NIFTI format and the first 10 time points for each file were removed. Second, the timing correction and realignment were carried out, the T1WI to the mean functional image was co-registered, and the DARTEL tool to compute transformations from individual native space to Montreal Neurological Institute (MNI) space was used (Yan et al., 2017). The subjects with head motion exceeding 2 mm or 2° were excluded. Then, the spatial smoothing using a 4-mm Gaussian kernel was performed and the low-frequency drift and high-frequency noise using band-pass filtering (0.01–0.1 Hz) were removed. Finally, the Friston 24-parameter model was used to regress out head motion effects. In this step, the white matter signal, cerebrospinal fluid signal, and global signal were regressed as covariates.

### Definition of the Region of Interest

**FIGURE 1:**
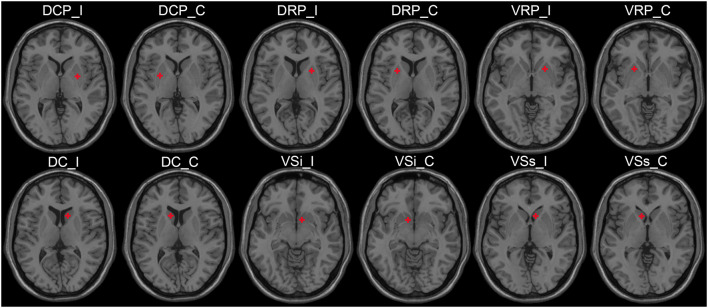
The ROI schematic diagrams of striatal subregions. ROI, region of interest; I, ipsilateral; C, contralateral; DCP, dorsal caudal putamen; DRP, dorsal rostral putamen; VRP, ventral rostral putamen; DC, dorsal caudate; VSi, inferior ventral striatum; VSs, superior ventral striatum.

The regions of interest (ROIs) were determined by the “Define ROI” module using DPARSF software, based on the radius and MNI spatial coordinates. According to the previous study, six striatal subregions of each hemisphere were selected as ROIs, and the radius of each ROI was set to 3 mm. The MNI coordinates of the ROIs were as follows: dorsal caudal putamen (DCP, ± 28, 1, 3), dorsal rostral putamen (DRP, ± 25, 8, 6), ventral rostral putamen (VRP, ± 20, 12, − 3), dorsal caudate (DC, ± 13, 15, 9), inferior ventral striatum (VSi, ± 9, 9, −8), and superior ventral striatum (VSs, ± 10, 15, 0) (Song et al., 2017). The diagram of ROIs is shown in Figure 1.

### Analysis of Functional Connectivity Based on Voxel-Wise

First, the time course of the average BOLD signal was extracted from each ROI. Then, the Pearson’s correlation between the time course of each ROI and the time course of all other voxels in the brain was calculated. Fisher *z* transformation was performed to improve the normal distribution of the data. Then, the correlation map, that is, the FC map of the whole brain was generated for further statistical analysis.

#### Intragroup Functional Connectivity Analysis

To explore the intragroup FC patterns of the cortical-striatal network, one-sample *t-*tests (default mask) were performed in the HFS group and the HC group. The statistical threshold was set at *z* > 3.6594 and cluster size of >6 mm^3^, which corresponded to a corrected *P* < 0.001. This correction threshold was determined using Monte Carlo simulations with the program AlphaSim in AFNI (Ledberg et al., 1998).

#### Between-Group Functional Connectivity Analysis

To determine the FC differences of the cortical-striatal network between the two groups, two-sample *t*-tests were performed at each FC map of 12 ROIs (default mask). The Gaussian random field (GRF) method was used for multiple comparison corrections, and the statistical threshold was set at voxel-level *P* < 0.005 and cluster-level *P* < 0.05. The DPABI software was used to perform the statistical analysis of FC.

#### Correlation Analysis

The *z* value of brain regions with significant changes in the HFS group was extracted to explore the relationship between the severity of HFS and altered connectivity. Then, the Spearman correlation analysis was performed using GraphPad Prism 6.0 software to evaluate the correlation between abnormal FC and spasm severity in patients with HFS. The age, sex, education, and duration were regressed as covariates. We also explored the relationship between duration and spasm severity through Spearman correlation analysis, and the age, sex, and education level were regressed as covariates as well. A total of 14 correlations were performed, with 13 abnormal FCs correlating with spasm severity, and, finally, 1 correlation was performed between spasm severity and disease duration. The false discovery rate (FDR) method was used to correct the results of the correlation analysis for multiple comparisons.

### Analysis of Functional Connectivity Based on Regions of Interest-Wise

The FC between the striatal ROIs was also calculated. After Fisher *z* transformation, a 12 × 12 FC matrix was generated for every subject. A total of 66 *z* values were constructed, and each *z* value stands for the FC between two brain regions. Two-sample *t*-tests were performed using SPSS 20.0 software (SPSS Inc., Chicago, IL, United States) to compare the difference of FC between the HFS group and the HC group. Age, gender, and education were regressed as covariates. FDR correction was used to control false positives for multiple comparisons, and the statistical threshold was set at *P* < 0.05.

## Results

### Clinical Results

There were no significant differences in sex, age, and education level between the HFS group and the HC group (*P* > 0.05) (Table 1).

### Intragroup Functional Connectivity in the Cortical-Striatal Network

**FIGURE 2:**
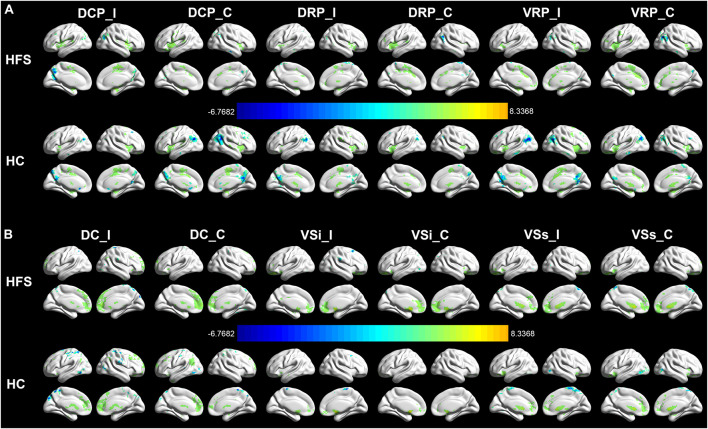
Intragroup FC in the cortical-striatal network. The FC maps of the cortical-striatal network in the HC group and HFS group were similar (AlphaSim correction, *P* < 0.001, cluster size > 6 mm^3^). **(A)** The putamen ROIs had strengthened connectivity with the insula, MCC, precuneus, and SMA. **(B)** The dorsal caudate ROIs had strengthened connectivity with the ACC and SFG. The ventral striatum ROIs had strengthened connectivity with ACC and OFC. The color bar represents the *t* value. FC, functional connectivity; HC, healthy control; HFS, hemifacial spasm; MCC, middle cingulate cortex; SMA, supplementary motor area; ACC, anterior cingulate cortex; SFG, superior frontal gyrus; OFC, orbitofrontal cortex; I, ipsilateral; C, contralateral; DCP, dorsal caudal putamen; DRP, dorsal rostral putamen; VRP, ventral rostral putamen; DC, dorsal caudate; VSi, inferior ventral striatum; VSs, superior ventral striatum.

The intragroup FC maps of the cortical-striatal network were similar in the HC and HFS groups, which is consistent with previous studies (Di Martino et al., 2008; Song et al., 2017; Dong et al., 2019). The putamen ROIs had strengthened connectivity with the insula, middle cingulate cortex (MCC), precuneus, and supplementary motor area (SMA) (Figure 2A). The DC ROIs had strengthened connectivity with the anterior cingulate cortex (ACC) and superior frontal gyrus (SFG). The ventral striatum ROIs had strengthened connectivity with ACC and OFC (Figure 2B). In this study, these intragroup maps were merely for visualizing FC in the two groups.

### Group Differences of Functional Connectivity in the Cortical-Striatal Network

#### Dorsal Caudal Putamen

**FIGURE 3:**
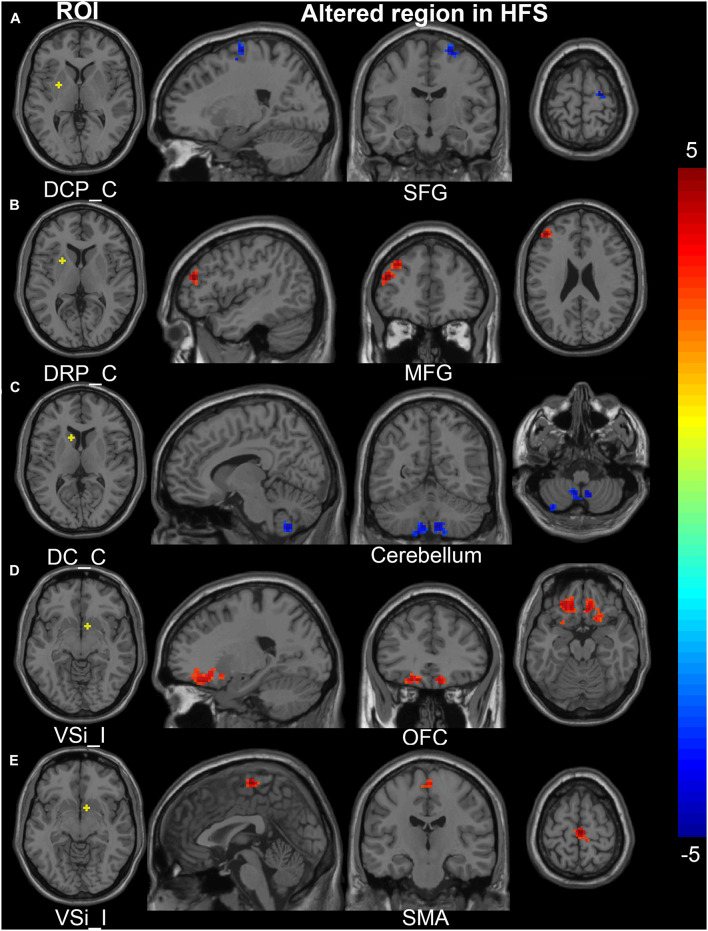
Altered FC in the cortical-striatal network (putamen, caudate, and inferior ventral striatum ROIs). In the HFS group compared with the HC group: **(A)** The FC between contralateral DCP and ipsilateral SFG was decreased. **(B)** The FC between contralateral DRP and contralateral MFG was increased. **(C)** The FC between contralateral DC and bilateral cerebellum was decreased. **(D)** and **(E)** The ipsilateral VSi showed increased FC with bilateral OFC and SMA. The color bar represents the *t* value. FC, functional connectivity; ROIs, regions of interest; HFS, hemifacial spasm; HC, healthy control; C, contralateral; I, ipsilateral; DCP, dorsal caudal putamen; DRP, dorsal rostral putamen; DC, dorsal caudate; VSi, inferior ventral striatum; SFG, superior frontal gyrus; MFG, middle frontal gyrus; OFC, orbitofrontal cortex; SMG, supramarginal gyrus; SMA, supplementary motor area.

**FIGURE 4:**
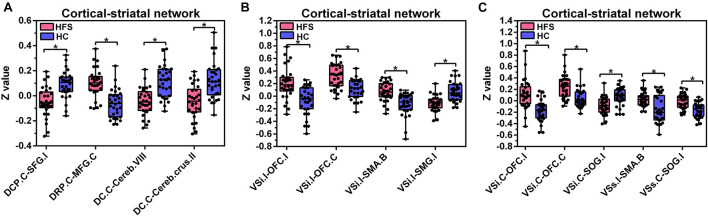
Altered FC in the cortical-striatal network. Boxplots with between-group differences for 13 significant cluster. In the HFS group compared with the HC group: **(A)** The FC between contralateral DCP and ipsilateral SFG was decreased. The FC between contralateral DRP and contralateral MFG was increased. The FC between contralateral DC and bilateral cerebellum was decreased. **(B)** The ipsilateral VSi showed increased FC with bilateral OFC and SMA. The FC between ipsilateral VSi and ipsilateral SMG was decreased. **(C)** The FC between contralateral VSi and bilateral OFC was increased. The FC between contralateral VSi and ipsilateral SOG was decreased. The FC between ipsilateral VSs and bilateral SMA was increased. The FC between contralateral VSs and ipsilateral SOG was increased. FC, functional connectivity; HFS, hemifacial spasm; HC, healthy control; C, contralateral; I, ipsilateral; B, bilateral; DCP, dorsal caudal putamen; SFG, superior frontal gyrus; DRP, dorsal rostral putamen; MFG, middle frontal gyrus; DC, dorsal caudate; Cereb, cerebellum; VSi, inferior ventral striatum; OFC, orbitofrontal cortex; SMA, supplementary motor area; SMG, supramarginal gyrus; SOG, superior occipital gyrus; VSs, superior ventral striatum.

The contralateral DCP had significantly decreased FC with ipsilateral SFG, SMA, and precentral gyrus, respectively, in the HFS group compared with the HC group (Figures 3A, 4A and Table 2).

**TABLE 2:** Altered FC in the putamen and caudate ROIs between two groups.

ROIs	Contrast	Brain region	Cluster size	Peak *t* value	MNI coordinates (mm)		
					x	y	z
DCP_C	HFS < HC	I superior frontal gyrus	55	–4.8035	21	−12	69
DRP_C	HFS > HC	C middle frontal gyrus	64	5.0707	−45	42	24
DC_C	HFS < HC	B cerebellum posterior lobe	165	–4.8075	9	−57	−51
	HFS < HC	C cerebellum crus2	76	–4.4086	−42	−75	−48

*FC, functional connectivity; ROIs, regions of interest; C, contralateral; DCP, dorsal caudal putamen; DRP, dorsal rostral putamen; DC, dorsal caudate; HFS, hemifacial spasm; HC, healthy control; MNI, Montreal Neurological Institute.*

#### Dorsal Rostral Putamen

The contralateral DRP had significantly increased FC with contralateral middle frontal gyrus (MFG) and SFG, in the HFS group compared with the HC group (Figures 3B, 4A and Table 2).

#### Ventral Rostral Putamen

There was no significant alteration in FC between VRP and cerebral cortex in the HFS group compared with the HC group.

#### Dorsal Caudate

The contralateral DC had significantly decreased FC with bilateral cerebellar lobule VIII, IX, and contralateral cerebellar crus II, in patients with HFS than that in controls (Figures 3C, 4A and Table 2).

#### Inferior Ventral Striatum

**FIGURE 5:**
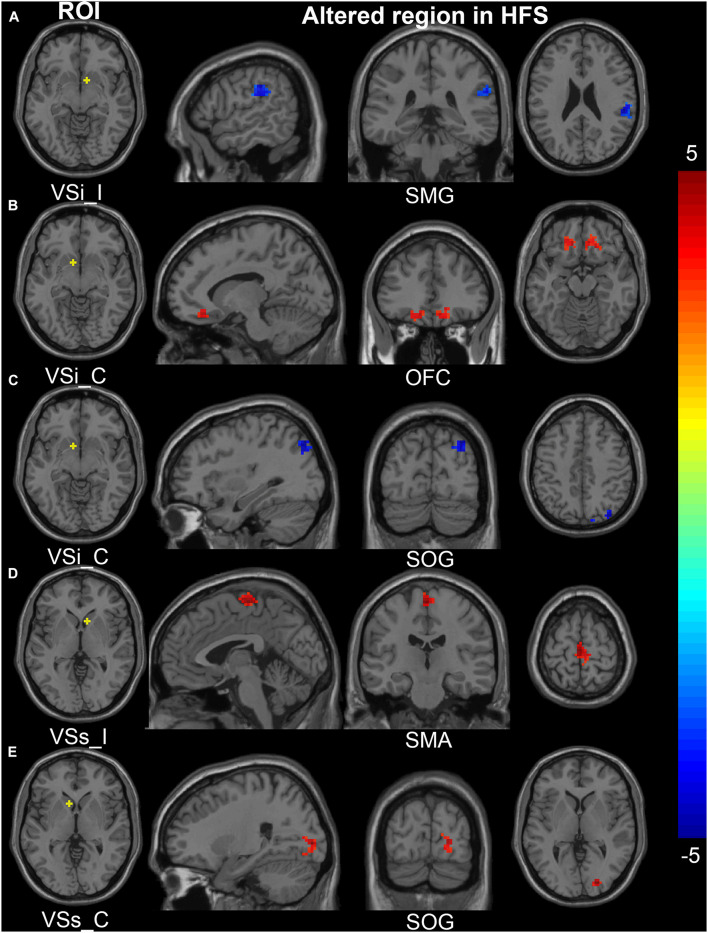
Altered FC in the cortical-striatal network (inferior and superior ventral striatum ROIs). In the HFS group compared with the HC group: **(A)** The FC between ipsilateral VSi and ipsilateral SMG was decreased. **(B)** The FC between contralateral VSi and bilateral OFC was increased. **(C)** The FC between contralateral VSi and ipsilateral SOG was decreased. **(D)** The FC between ipsilateral VSs and bilateral SMA was increased. **(E)** The FC between contralateral VSs and ipsilateral SOG was increased. The color bar represents the *t* value. FC, functional connectivity; ROIs, regions of interest; HFS, hemifacial spasm; HC, healthy control; I, ipsilateral; C, contralateral; VSi, inferior ventral striatum; VSs, superior ventral striatum; SMG, supramarginal gyrus; OFC, orbitofrontal cortex; SOG, superior occipital gyrus; SMA, supplementary motor area.

In the HFS group compared with the HC group, the ipsilateral VSi had significantly increased FC with bilateral OFC, paracentric lobule, and SMA (Figures 3D,E, 4B and Table 3), while the FC between ipsilateral VSi and superior marginal gyrus (SMG) was significantly decreased (Figures 4B, 5A and Table 3); the FC between contralateral VSi and bilateral OFC was significantly increased (Figures 4C, 5B and Table 3), while the FC between contralateral VSi and ipsilateral superior occipital gyrus (SOG) was significantly decreased (Figures 4C, 5C and Table 3).

**TABLE 3:** Altered FC in the ventral striatum ROIs between two groups.

ROIs	Contrast	Brain region	Cluster size	Peak *t* value	MNI coordinates (mm)		
					x	y	z
VSi_I	HFS > HC	I orbitofrontal cortex	107	4.681	9	30	−18
	HFS > HC	C orbitofrontal cortex	166	4.4424	−21	33	−18
	HFS > HC	B supplementary motor area	56	5.0717	3	−21	66
	HFS < HC	I supramarginal gyrus	71	-5.2399	54	−30	24
VSi_C	HFS > HC	I orbitofrontal cortex	94	4.6397	9	27	−24
	HFS > HC	C orbitofrontal cortex	58	4.0489	−12	36	−15
	HFS < HC	I superior occipital gyrus	71	-4.3179	33	−78	45
VSs_I	HFS > HC	B supplementary motor area, paracentral lobe	76	4.534	−3	−21	66
VSs_C	HFS > HC	I superior occipital gyrus	59	4.629	21	−87	3

*FC, functional connectivity; ROIs, regions of interest; I, ipsilateral; C, contralateral; VSi, inferior ventral striatum; VSs, superior ventral striatum; HFS, hemifacial spasm; HC, healthy control; MNI, Montreal Neurological Institute.*

#### Superior Ventral Striatum

The ipsilateral VSs showed significantly increased FC with bilateral SMA and paracentric lobule, in the HFS group compared with the HC group (Figures 4C, 5D and Table 3). The FC between contralateral VSs and ipsilateral SOG in patients with HFS was significantly increased than that in controls (Figures 4C, 5E and Table 3).

### Correlation Between Spasm Severity and Functional Connectivity and Duration

**FIGURE 6:**
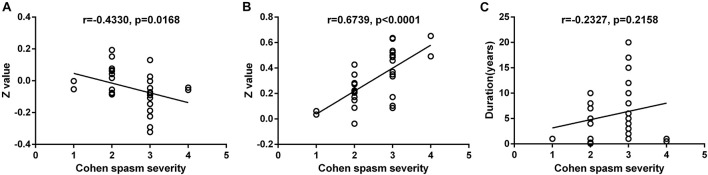
The correlation relationship between spasm severity and altered FC and duration. **(A)** The FC between contralateral DCP and ipsilateral SFG was negatively correlated with the Cohen spasm scores (*r* = −0.433, *P* = 0.0168 uncorrected). **(B)** The FC between ipsilateral VSi and contralateral OFC showed positive correlation with the Cohen spasm scores (*r* = 0.6739, *P* < 0.0001 uncorrected). **(C)** There was no correlation between the duration and the spasm severity (*r* = − 0.2327, *P* = 0.2158 uncorrected). FC, functional connectivity; DCP, dorsal caudal putamen; SFG, superior frontal gyrus; VSi, inferior ventral striatum; OFC, orbitofrontal cortex.

The FC between contralateral DCP and ipsilateral SFG was negatively correlated with the Cohen spasm scores (*r* = −0.433, *P* = 0.0168 uncorrected) (Figure 6A). Furthermore, the FC between ipsilateral VSi and contralateral OFC showed positive correlation with the Cohen spasm scores (*r* = 0.6739, *P* < 0.0001 uncorrected) (Figure 6B). There was no correlation between the duration and the spasm severity (*r* = −0.2327, *P* = 0.2158 uncorrected) (Figure 6C). After FDR correction, there was no significant correlation between abnormal FCs and spasm severity.

### Within the Striatal Network

**FIGURE 7:**
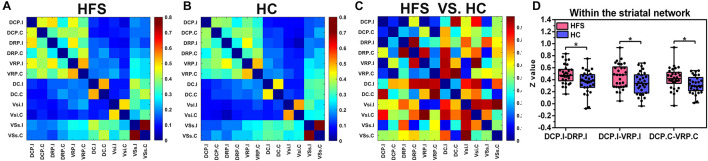
The FC maps of the striatal network. **(A)** and **(B)** The FC matrix map of the striatal network in the HFS group and the HC group. The color bar represented the mean *z* value. **(C)** The group differences of the FC within the striatal network between the two groups. The color bar represents the *P* value. **(D)** In the HFS group compared with the HC group: The ipsilateral DCP showed increased FC with ipsilateral DRP and VRP. In addition, the FC between contralateral DCP and contralateral VRP was increased. * indicates *P* < 0.05 (uncorrected). FC, functional connectivity; HFS, hemifacial spasm; HC, healthy control; DCP, dorsal caudal putamen; DRP, dorsal rostral putamen; VRP, ventral rostral putamen.

Compared with the HC group, the ipsilateral DCP in the HFS group showed increased FC with ipsilateral DRP and VRP (*P* = 0.0053, *P* = 0.0272 uncorrected), and the FC between contralateral DCP and contralateral VRP was also increased (*P* = 0.017 uncorrected) (Figure 7). After FDR correction, there was no significant difference in FC within the striatal network between the HFS group and the HC group.

## Discussion

This study investigated the functional alterations of the cortical-striatal network in patients with HFS and their relationship with clinical manifestations. Compared with the controls, the striatal subregions had altered FC with motor and OFC in patients with HFS. Furthermore, the FC between the ventral striatum and motor cortex was positively associated with the severity of HFS. Finally, our results suggest that the cortical-striatal network may play differential roles in the underlying pathological mechanism of HFS.

### Increased Functional Connectivity of the Cortical-Striatal Network in Patients With Hemifacial Spasm

As we know, this is the first resting-state fMRI study to examine intrinsic cortical-striatal connectivity in HFS. The emotion-related cortex showed significantly increased FC with the ventral striatum and putamen, in patients with HFS compared with the controls. The orbitofrontal lobe and the VSi are involved in emotional activities (Di Martino et al., 2008; Accolla et al., 2016). The structural and functional abnormalities of those regions were found to be widespread in patients with depression (Botteron et al., 2002; Bremner et al., 2002; Taylor et al., 2003; Ballmaier et al., 2004). Long-term HFS may lead to anxiety and depression (Bao et al., 2015). The increased connectivity between the VSi and orbitofrontal lobe may be associated with the poor mental status of patients with HFS. The SOG was located in the visual network, and studies have found that this area was involved in facial expression and emotion processing (Tao et al., 2013). We speculated that the increased functional activity of these regions in patients with HFS may be related to abnormal facial expressions. In addition, the putamen had increased FC with MFG and SFG, which may be associated with the depression and other adverse emotions of the patients with HFS, and it is consistent with previous studies in patients with depression (Fitzgerald et al., 2006). In summary, the ventral striatum was mainly involved in emotional activities, while the putamen may also involve in emotional activities in addition to motor function.

### Decreased Functional Connectivity of the Cortical-Striatal Network in Patients With Hemifacial Spasm

We also found that the FC between the putamen and the ipsilateral motor cortex was decreased in the HFS group, and so does the FC between the caudate and the cerebellum. It was known that the precentral gyrus was the first somatic motor area, and the SFG was the premotor area. One side of the cerebral motor area dominates the contralateral body movement, but the muscles involved in associated movement are dominated by bilateral motor areas, such as extraocular muscles and masticatory muscles (Desai et al., 2013). The decrease of FC between the putamen and ipsilateral motor area may be a compensatory mechanism to inhibit facial muscle spasms. In addition, the cerebellum is an important motor regulation center. The cerebellum may be involved in the processing of movement, cognition, and emotion by forming loops with the brain, and structural or functional abnormalities in these loops may contribute to the development of motor disorders (e.g., ataxia) (D’Angelo and Casali, 2013). Abnormalities in the connectivity between brain regions within the loops can lead to disorders that are associated with loop dysfunction. The cerebellum may form a loop with the cortical-caudate, which is involved in the motor regulation of HFS, and the diminished connectivity may be a consequence of the dysfunction of the caudate-cerebellar loop. To sum up, in addition to the putamen, other parts of the striatum, such as the caudate, may be involved in the pathological process of HFS. Different cortical-striatal loops may be involved in motion monitoring, error detection, and correction (Song et al., 2017).

### Increased Functional Connectivity Within the Striatal Network in Patients With Hemifacial Spasm (Uncorrected, *P* < 0.05)

In this study, we found no significant differences in FC within the striatal network between the two groups (FDR correction, threshold *P* < 0.05). However, using a less conservative threshold (*P* < 0.05), we found that the FC between the putamen ROIs was increased in patients with HFS compared with the controls. Interestingly, a study of the striatal network in facial palsy found the decreased FC between the putamen and the ventral striatum (uncorrected) (Song et al., 2017), which is the opposite of our results. We know that facial palsy and facial spasm are two motor disorders with opposite manifestations, the former shows reduced or no movement of the affected facial muscles and the latter shows excessive movement. In addition, the connectivity within the striatal network is also consistent with clinical manifestations, i.e., being diminished in facial palsy and increased in facial spasm. Therefore, we speculated that the putamen plays an important role in the motor function of facial muscles, and the increased or decreased FC between the putamen and other seeds within the striatum may respond to the increased or decreased motor signals in this neural circuit, thus resulting in different motor states of the facial muscles.

### Limitations

There were several limitations in this study. First, the sample size of this study was small, including patients with left HFS and right HFS. To control the problem on the different sides of the lesion, we flipped the left HFS group from left to right. In the future, the changes of brain functional networks in patients with left and right HFS can be studied separately on the basis of expanding the sample size. Second, this study used a cross-sectional study design to explore the changes of the cortical-striatal network in patients with HFS. Longitudinal studies can be conducted in the future to explore the mechanism of brain network changes in different stages of HFS. Finally, previous studies and our study have all found functional abnormalities in the emotion-related cortex in patients with HFS. Therefore, increasing the evaluation of the psychological status of patients may make the results more reliable, which can be added in further studies.

## Conclusion

Primary unilateral HFS induces several FC alterations in the cortical-striatal network, specifically, the striatal subregions have altered connectivity with motor and OFC in patients with HFS, respectively. The severity of HFS is associated with these functional alterations. This study provides significant evidence that the altered cortical-striatal connectivity is involved in differential neural mechanisms of motor and emotional dysfunction in patients with HFS.

## Data Availability Statement

The original contributions presented in the study are included in the article/supplementary material, further inquiries can be directed to the corresponding author.

## Ethics Statement

The studies involving human participants were reviewed and approved by the Ethics Committee of China-Japan Friendship Hospital. The patients/participants provided their written informed consent to participate in this study.

## Author Contributions

GM conceived and designed the experiments. WG and DY performed the experiments. WG and ZZ analyzed the data. WG, LD, BL, JL, YC, YW, XL, AY, KL, and JX discussed the data. WG wrote the manuscript. DY revised the manuscript. All authors read and approved the final manuscript.

## Conflict of Interest

The authors declare that the research was conducted in the absence of any commercial or financial relationships that could be construed as a potential conflict of interest.

## Publisher’s Note

All claims expressed in this article are solely those of the authors and do not necessarily represent those of their affiliated organizations, or those of the publisher, the editors and the reviewers. Any product that may be evaluated in this article, or claim that may be made by its manufacturer, is not guaranteed or endorsed by the publisher.
